# Association of body composition with bone mineral density and fractures in Chinese male type 2 diabetes mellitus

**DOI:** 10.1097/MD.0000000000033400

**Published:** 2022-04-07

**Authors:** Chuchen Meng, Dan Zhao, Xin-Hua Ye

**Affiliations:** a Department of Endocrinology, The Affiliated Changzhou No.2 People’s Hospital of Nanjing Medical University, Changzhou, Jiangsu, China.

**Keywords:** bone mineral density, fat mass, lean mass, osteoporotic fracture, type 2 diabetes mellitus

## Abstract

The association between body composition and bone health in men over 50 years with type 2 diabetes mellitus remains unclear. We aimed to investigate how fat and lean mass affect bone health in male patients with diabetes over 50 years. A total of 233 hospitalized male type 2 diabetes mellitus patients with aged 50 to 78 years were enrolled. Lean mass, fat mass and bone mineral density (BMD) were estimated. The clinical fractures were also assessed. Glycosylated hemoglobin, bone turnover markers, and biochemical parameters were measured. The normal BMD group had a higher lean mass index (LMI) and fat mass index (FMI) and lower levels of bone turnover markers. glycosylated hemoglobin was negatively correlated with LMI (r = −0.224, *P* = .001) and FMI (r = −0.158, *P* = .02). In partial correlation adjusted for age and body weight, FMI was negatively correlated (r = −0.135, *P* = .045) with lumbar spine, while LMI was still positively correlated with lumbar spine (*R* = 0.133, *P* = .048) and total hip (*R* = 0.145, *P* = .031). In multiple regression analysis, LMI was consistently associated with BMD at the spine (β = 0.290, *P* < .01), hip (β = 0.293, *P* < .01), and femoral neck (β = 0.210, *P* = .01), whereas FMI was only positively associated with BMD at the femoral neck (β = 0.162, *P* = .037). A total of 28 patients diagnosed with diabetic osteoporotic fractures had lower LMI and FMI than their non-fractured counterparts. LMI was negatively associated with fracture, whereas FMI had such an effect only before adjusting for BMD. Lean mass is dominant in maintaining BMD and is a BMD-independent protective factor for diabetic osteoporotic fracture in male patients aged over 50 years. Fat mass in gravity is positively associated with BMD in the femoral neck, which may mediate fracture protection.

## 1. Introduction

Type 2 diabetes mellitus (T2DM) is a highly prevalent metabolic disorder. One of its most common comorbidities is osteoporosis, characterized by low bone mass and weakened bone microstructure, resulting in reduced bone strength and increased fracture risk, especially in patients over 50 years of age. The association between T2DM and bone health is complex. Despite the reported higher bone mineral density (BMD) compared to the healthy population.^[1]^ T2DM patients have an increased risk of fracture.^[2,3]^ T2DM is frequently accompanied by overweight or obesity, which play a key role in bone health. Historically, increased body weight was thought to protect against osteoporosis or fracture. ^[4–6]^ However, recent studies have challenged this belief. A UK study in 2011 reported that obesity was not protective against fractures in postmenopausal women.^[7]^ Another study involving severely obese women found that body mass index (BMI) ≥ 50 kg/m^2^ was a risk factor for lower BMD.^[8]^ A cross-sectional study showed that both overweight and underweight were risk factors for vertebral fracture in T2DM patients, independent of BMD.^[9]^ We noticed in most studies that obesity was assessed by BMI, which is commonly used in clinical practice and can effectively indicate diseases such as cardiovascular diseases and gout.^[10]^ Nevertheless, it may not assess the overall body composition, as the proportions of fat and lean mass can vary noticeably, even with the same BMI. Therefore, analogous to BMI, (fat mass index [FMI], fat mass in kg/height in m^2^), and (lean mass index [LMI], lean mass in kg/height m^2^) are suggested to be more accurate indicators of body composition.

In the general population, several studies have demonstrated that increased lean mass rather than fat mass is associated with bone mass.^[11–13]^ However, some studies have suggested that both lean and fat mass play a key positive role in the BMDs of young and middle-aged adults.^[14,15]^ Some studies have also shown that fat mass adversely affects bone mass independently.^[16,17]^ Although the conclusions of fat mass were not completely consistent, which may be due to a wide variety of age, ethnicity, and sex, most studies believe that lean mass plays a leading role in maintaining bone mass.

It has been reported that T2DM patients have a higher fat mass and lower lean mass level than healthy control,^[18–20]^ and whether body composition plays the same role in T2DM is indistinct. Investigations of the effects of body composition on bone health have long focused on the general population, especially in pre- or postmenopausal women, while male diabetic patients have been underestimated.

Consequently, we aimed to explore the relationship between FMI, LMI, BMD values, and clinical fractures in male patients with type 2 diabetes to provide theoretical evidence for maintaining bone health.

## 2. Materials and methods

### 2.1. Study subjects

The study was performed on patients with type 2 diabetes who were admitted to the Affiliated Changzhou No.2 People’s Hospital of Nanjing Medical University between January 2017 and May 2018. The study protocol was reviewed and approved by the ethics committee of the Affiliated Changzhou No.2 People’s Hospital of Nanjing Medical University. The selected patients were in line with the World Health Organization diagnostic criteria for type 2 diabetes and were restricted to over 50-year-old men. Exclusion criteria were type 1 diabetes; history of anti-osteoporosis medication; use of drugs that might influence bone metabolism for more than 6 months or within the previous 12 months, such as thiazolidinediones, immunosuppressants, systemic glucocorticoids, and hormone replacement therapy; active hepatitis/liver cirrhosis; chronic renal failure; congestive heart failure; malignant tumors; parathyroid dysfunction; Cushing syndrome; abnormal thyroid function; and other diseases that may affect bone metabolism. A total of 233 subjects, aged 50 to 78 years, were included in the study. Demographic information was obtained during health interviews. All participants were asked about the occurrence of clinical fractures, including the time, site, and cause of the fractures.

### 2.2. Laboratory tests

BMI was calculated by dividing body weight by height squared (kg/m^2^). Serum calcium (Ca) and phosphorous (P) were measured enzymatically using a Cobas8000 modular analyzer series (Roche Diagnostics GmbH, Mannheim, Germany). Glycosylated hemoglobin (HbA1c) levels were measured using HPLC (HLC-723G7; Tosoh Co., Tokyo, Japan). Urinary albumin-to-creatinine ratio was measured (DCA 2000 + microalbuminuria Analyzer; Bayer Diagnostics, Tarrytown, NY). The amino-terminal propeptide of type 1 procollagen (TP1NP), cross-linked C-telopeptide (β-CTX), 25-hydroxyvitamin D, and parathyroid hormone levels were determined using an automated cobase601 Roche electrochemiluminescence system (Roche Diagnostics Gmbh, Germany).

### 2.3. BMD and body composition measurements

Lumbar spine L1-L4, femoral neck, and left total hip were measured using EXPLORER dual-energy X-ray absorptiometry (HOLOGIC, Bedford, MA). BMD data obtained from DXA were expressed as grams per centimeter squared (g/cm^2^). Osteoporosis was defined in subjects with a T-score of≦−2.5 standard deviation (SD), and osteopenia was diagnosed by a − 2.5 < T-score < −1.0 SD according to the criteria established by the World Health Organization. Quality control was performed daily before the self-test, and the instrument measurement coefficient of variation was <1%. Lean and fat masses were obtained from whole-body DXA scans. LMI = total body lean mass/height2 (kg/m^2^); FMI = total body fat mass/height2 (kg/m^2^).

### 2.4. Complications and osteoporotic fracture

Osteoporotic fracture is diagnosed when the following 2 conditions are met: Osteoporosis or osteopenia; and Fracture occurring in the hip, femoral neck, lumbar spine, or forearm caused by minimal or moderate trauma.

Diabetic nephropathy was defined as a urinary albumin-to-creatinine ratio ≥ 30 mg/g at 2 of 3 consecutive measurements, while other possible causes of proteinuria were excluded. Diabetic retinopathy was diagnosed using fundus photography or fluorescein angiography. Patients who complained of bilateral sensory deterioration on the toes and plantar, weakened or absent bilateral Achilles tendon reflex, and bilateral vibration sensation in the medial malleolus were diagnosed as diabetic peripheral neuropathy (DPN). Alcohol consumption was categorized as drinking 2 units of alcohol per day for at least 5 years. Current smokers were defined as current smokers with a lifetime smoking history of 300 cigarettes.

### 2.5. Statistical analyses

Data analysis was performed using SPSS version 17.0 for Windows (Chicago, IL). Mean ± SD was calculated as a numerical variable. Normally distributed variables are expressed as the mean ± SD. When comparing continuous variables, the student *t* test was used for normally distributed data. The chi-squared test of independence or Fisher exact test was used to test the distribution of categorical variables. The correlation between BMD, body composition, and other clinical parameters was analyzed by partial correlation adjusted for age. The influence of body weight was eliminated when further studying the correlation between BMD and FMI and LMI. Multiple linear regression was used to assess the relationships between the FMI, LMI, and BMD at different sites. Multivariable logistic regression models were used to estimate the odds ratios (OR) for clinical fractures. Statistical significance was set at *P* < .05.

## 3. Results

Among the male diabetic participants who were over 50 years old from Changzhou No.2 Hospital, 34 were diagnosed with osteoporosis and 91 had osteopenia. The baseline characteristics of the patients are shown in Table 1.

**Table 1:** Baseline comparative data of diabetic patients with normal BMD, osteopenia and osteoporosis.

	Normal (n = 98)	Osteopenia (n nt91)	Osteoporosis (n nt34)	*P* value
BMI (kg/m2)	25.93 ± 2.79	24.56 ± 2.59a	23.65 ± 3.46 a, b	<.001
Age (yr)	60.73 ± 6.90	62.18 ± 6.92	60.71 ± 6.35	.299
Duration of diabetes (yr)	9.06 ± 6.72	9.72 ± 6.53	8.75 ± 7.49	.707
HbA1c (%)	9.63 ± 2.24	9.78 ± 2.19	9.86 ± 2.19	.842
PTH (ng/L)	35.74 ± 20.22	35.41 ± 20.42	43.48 ± 21.58	.120
25-(OH) D (nmol/L)	45.42 ± 15.38	50.75 ± 65.39	44.14 ± 16.97	.669
β-CTX (pg/mL)	186.67 ± 95.67	198.57 ± 93.92 a	271.69 ± 145.76 a, b	.001
TP1NP (ng/mL)	27.01 ± 10.64	28.54 ± 12.15	35.62 ± 14.40 a, b	.003
Calcium (mmol/L)	2.27 ± 0.29	2.24 ± 0.13	2.24 ± 0.09	.621
Phosphorus(mmol/L)	1.15 ± 0.14	1.14 ± 0.20	1.16 ± 0.12	.818
Smoking n (%)	48 (48.98)	44 (48.35)	24 (70.59)	.062
Alcohol use n (%)	18 (19.57)	22 (24.18)	8 (23.53)	.595
Hypoglycemic agents n (%)				
Metformin	28 (28.57)	29 (31.87)	12 (35.29)	.742
Insulin	34 (34.69)	36 (39.56)	10 (29.41)	.545
DPP-4 inhibitor	1 (1.02)	5 (5.49)	2 (5.88)	.188
SGLT-2 receptor agonist	1 (1.02)	0	0	.527
Alpha-glucosidase inhibitor	28 (28.57)	20 (26.37)	11 (32.35)	.539
Sulfonylurea	37 (37.76)	24 (26.37)	9 (26.47)	.193
Microvascular complications n (%)				
DR	21 (21.43)	22 (24.18)	6 (17.65)	.724
DN	13 (13.27)	16 (17.58)	4 (11.76)	.609
DPN	61 (62.2)	68 (74.7)	22 (64.7)	.171
BMDs at different sites (kg/m3)				
Lumbar spine	1.11 ± 0.12	0.98 ± 0.09a	0.84 ± 0.12 a, b	<.001
Femoral neck	0.89 ± 0.11	0.76 ± 0.10a	0.68 ± 0.12 a, b	<.001
Total hip	1.04 ± 0.11	0.92 ± 0.09a	0.83 ± 0.13 a, b	<.001
Body composition parameters				
FMI (kg/m2)	7.22 ± 1.64	6.56 ± 1.57a	6.01 ± 1.69 a	<.001
LMI (kg/m2)	16.97 ± 1.44	16.47 ± 1.72a	16.08 ± 1.86 a	.012
Fracture incidence n (%)	0	17 (18.68)	11 (32.35)	<.001

a: VS normal < 0.05; b VS osteopenia < 0.05.

25-(OH)D = 25-hydroxyvitamin D, β-CTX = β-isomerized C-terminal telopeptides, BMD = bone mineral density, BMI = body mass index, DN = diabetic nephropathy, DPN = diabetic peripheral neuropathy, DPP-4 = dipeptidyl peptidase-4, DR = diabetic retinopathy, FMI = fat mass index, HbA1c = hemoglobin A1c, LMI = lean mass index, PTH = parathyroid hormone, SGLT-2 = sodium-glucose cotransporter-2, TP1NP = total procollagen type 1 amino-terminal propeptide.

There were no significant differences in age, HbA1c, disease course, serum Ca, P, and 25-hydroxyvitamin D levels, incidence of microvascular complications, and hypoglycemic medication among the 3 groups. Compared with those with osteopenia and osteoporosis, patients with normal BMD had higher levels of BMI, LMI, and FMI, and lower levels of β-CTX and TP1NP (*P* < . 05). Patients with osteoporosis were more prone to fractures than those with osteopenia (*P* < . 001).

BMD values at the lumbar spine, femoral neck, and total hip were positively correlated with BMI, LMI, and FMI, and negatively correlated with β-CTX and TP1NP in partial correlation adjusted for age (*P* < .01, or *P* < .05). When the mechanical loading effect of body weight on BMD was further adjusted, the positive correlations between BMD values of the lumbar spine and FMI became negative (r = −0.135, *P* = .045), while those between the lumbar spine (*R* = 0.133, *P* = .048), total hip (*R* = 0.145, *P* = .031), and LMI remained positive (Table 2).

**Table 2:** Partial correlation between BMD, body composition and other biochemical and clinical parameters.

	Lumbar spine	Femoral neck	Total hip
	r	r	r
Course of diabetes (yr)	0.041	0.056	0.046
HbA1c (%)	−0.087	−0.061	−0.058
β-CTX (pg/mL)	−0.301**	−0.207*	−0.267**
TP1NP (ng/mL)	−0.265**	−0.168*	−0.254**
25 (OH) D (nmol/L)	−0.013	−0.010	−0.003
PTH (ng/L)	−0.057	−0.003	0.005
FMI (kg/m^2^)	0.197** (−0.135*)	0.221** (−0.056)	0.262** (−0.028)
LMI (kg/m^2^)	0.344** (0.133*)	0.247** (0.070)	0.328** (0.145*)

The value of regression coefficients adjusted for body weight were shown in parentheses.

25 (OH)D = 25-hydroxyvitamin D, β-CTX = β-isomerized C-terminal telopeptides, BMD *=* bone mineral density, FMI = fat mass index, HbA1c = hemoglobin A1c, LMI = lean mass index, PTH = parathyroid hormone, TP1NP = total procollagen type 1 amino-terminal propeptide.

* *P* < .05.

** *P* < .01.

**Figure 1.**
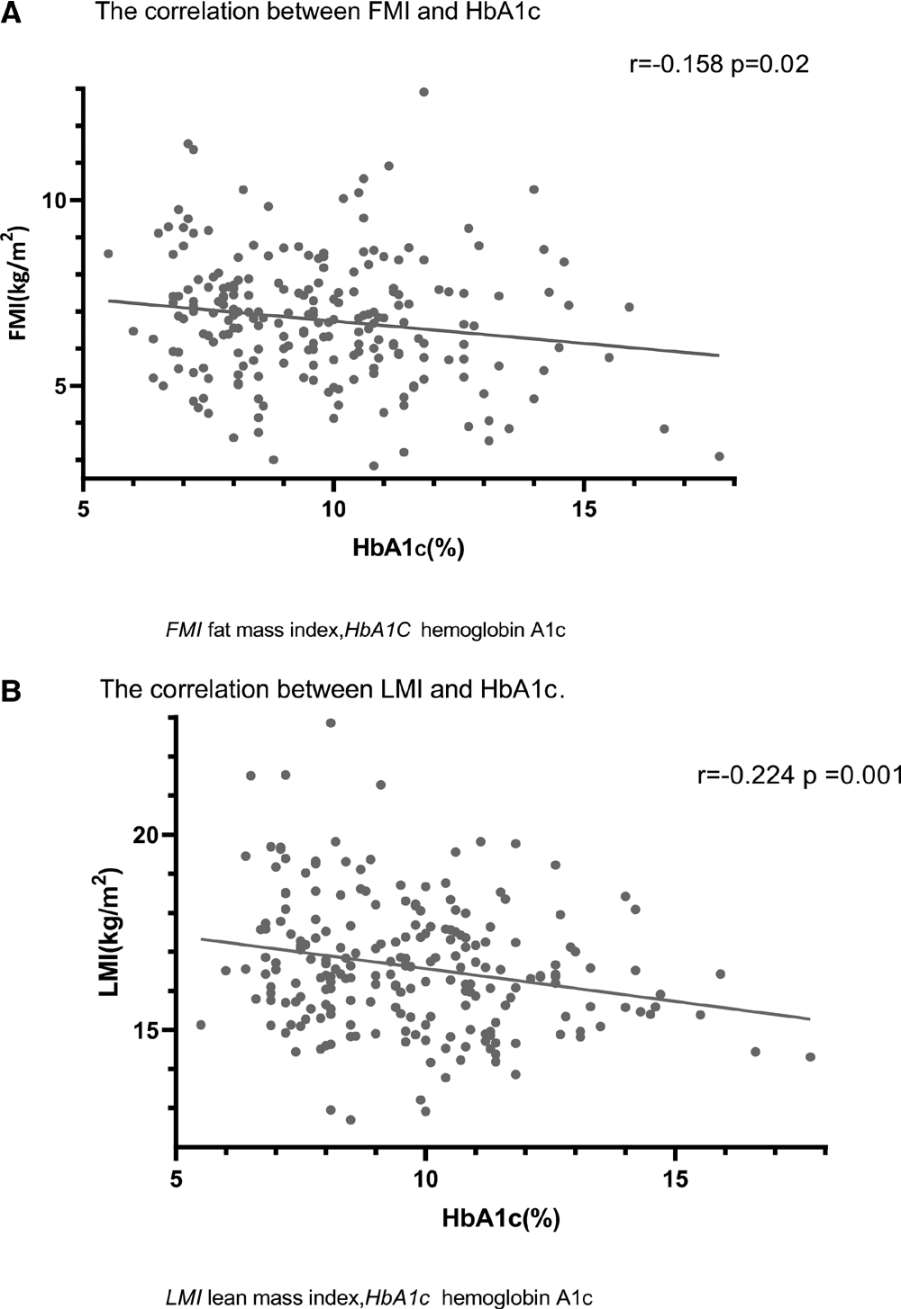
(A) The correlation between FMI and HbA1c and (B) The correlation between LMI and HbA1c; Each gray point represents a sample. The solid line represents the curve fit between variables. HbA1c = glycosylated hemoglobin, LMI = lean mass index, FMI = fat mass index.

HbA1c was negatively correlated with both FMI (r = −0.158 *P* = .02) and LMI (r = −0.224 *P* = .001) for bivariate correlation. (Fig. 1A and B)

To investigate the independent factors of BMD, multiple linear regression analyses were performed with age, years of diabetes course, HbA1c, FMI, LMI, diabetic complications, smoking, and alcohol use status as independent variables in different models (Table 3). LMI was consistently associated with BMD of the spine (β = 0.290, *P* < . 01), total hip (β = 0.293 *P* < . 01) and femoral neck (β = 0.210 *P* = .01). FMI was only positively associated with BMD of the femoral neck (β = 0.162, *P* = .037) after adjusting for all confounding factors.

**Table 3:** Multiple regression analysis of the data on FMI and LMI with BMD at different sites.

	Lumbar spine		Femoral neck		Total hip	
	*β*	*P* value	*β*	*P* value	*β*	*P* value
Model 1						
LMI	0.243	.001	0.234	.002	0.293	<.001
FMI	0.070	.349	0.126	.090	0.115	.116
Model2						
LMI	0.278	.001	0.193	.015	0.256	.001
FMI	0.051	.509	0.166	.031	0.148	.050
Model3						
LMI	0.290	<.001	0.210	.010	0.293	<.001
FMI	0.047	.550	0.162	.037	0.129	.088

Model 1 -unadjusted. Model 2-adjusted for diabetes duration, age and HbA1c. Model 3- adjusted for factors listed in Model 2 plus diabetic microvascular complications (diabetic retinopathy, diabetic nephropathy, diabetic peripheral neuropathy), smoking and alcohol use.

FMI = fat mass index, HbA1c = hemoglobin A1c, LMI = lean mass index.

We compared features of body composition parameters between diabetic patients with and without fractures. Twenty-eight of the patients were diagnosed with osteoporotic fractures. When comparing the body composition parameters and clinical and biochemical parameters, patients with diabetic osteoporotic fractures were significantly older and had lower BMI, LMI, FMI, and BMD values at the lumbar spine, femoral neck, and hip (*P* < .05). Patients with fractures had a higher prevalence of DPN, but there was no significant difference in the occurrence of diabetic nephropathy and diabetic retinopathy (Table 4).

**Table 4:** Comparison of body composition, biochemical and clinical parameters between patients with and without clinical fractures.

	Fracture (+) N = 195	Fracture (−) N = 28	*P* value
BMI (kg/m^2^)	25.41 ± 2.80	22.32 ± 2.47	<.001
Age (yr)	60.73 ± 6.65	65.39 ± 6.86	.001
Duration of diabetes (yr)	8.96 ± 6.48	11.46 ± 8.17	.127
HbA1C (%)	9.41 ± 2.61	9.97 ± 2.86	.296
PTH (ng/L)	37.37 ± 20.66	32.76 ± 20.24	.270
25-OH-VD (nmol/L)	44.88 ± 14.59	63.96 ± 14.67	.413
β-CTX (pg/mL)	198.94 ± 102.72	259.36 ± 137.87	.059
TP1NP (ng/mL)	28.32 ± 11.38	34.94 ± 16.93	.088
Ca (mmol/L)	2.25 ± 0.22	2.22 ± 0.13	.467
P (mmol/L)	1.15 ± 0.17	1.13 ± 0.12	.546
Smoking n (%)	105 (53.84)	11 (39.29)	.162
Alcohol use n (%)	45 (23.08)	3 (10.72)	.217
Hypoglycemic agents n (%)			
Metformin	61 (31.28)	8 (28.57)	.831
Insulin	70 (35.90)	10 (36.41)	.985
DPP4-I	6 (3.00)	2 (0.07)	.265
SGLT2-i	1 (0.01)	-	
Alpha-glucosidase inhibitor	51 (26.15)	9 (32.14)	.028
Sulfonylurea	63 (32.31)	7 (25.00)	.518
Microvascular complications n (%)			
DR	42 (21.54)	7 (25.00)	.633
DN	28 (14.36)	5 (17.86)	.577
DPN	127 (65.13)	24 (85.71)	.031
BMDs at different sites (kg/m^3^)			
Lumbar spine	1.03 ± 0.14	0.90 ± 0.15	<.001
Femoral neck	0.82 ± 0.15	0.67 ± 0.09	<.001
Total hip	0.98 ± 0.12	0.82 ± 0.12	<.001
Body composition parameters			
FMI (kg/m^2^)	6.93 ± 1.64	5.63 ± 1.42	.001
LMI (kg/m^2^)	16.85 ± 1.58	15.06 ± 1.28	<.001

25-(OH)D = 25-hydroxyvitamin D, β-CTX = β-isomerized C-terminal telopeptides, BMD = bone mineral density, BMI = body mass index, DN = diabetic nephropathy, DPN = diabetic peripheral neuropathy, DPP-4 = dipeptidyl peptidase-4, DR = diabetic retinopathy, FMI = fat mass index, HbA1c = hemoglobin A1c, LMI = lean mass index, PTH = parathyroid hormone, SGLT-2 = sodium-glucose cotransporter-2, TP1NP = total procollagen type 1 amino-terminal propeptide.

In multivariate logistic regression analysis (Table 5), when age was adjusted, both FMI and LMI were significantly associated with the presence of clinical fractures [OR = 0.485, 95% confidence interval (CI) = 0.443–0.979, *P* = .039, OR = 0.409, 95% CI = 0.307–0.767, *P* = .002] (Model 1). The association between FMI and LMI and the presence of clinical fractures still existed when further adjusted for age, diabetes duration, HbA1c, smoking, alcohol use, and the presence of diabetic complications (OR = 0.653, 95% CI = 0.430–0.991, *P* = .045; OR = 0.480, 95% CI = 0.297–0.774, *P* = .003, respectively) (Model 2). FMI was not statistically significant when BMD values at the 3 sites were considered in Model 3 (OR = 0.745, 95% CI = 0.444–1.251, *P* = .299), while the association between LMI and clinical fractures was not affected (OR = 0.450, 95% CI = 0.311–0.940, *P* = .029).

**Table 5:** Associations between the presence of clinical fractures and FMI, LMI in diabetic patients.

	Exp (B)	OR (95% CI)	*P* value
Model 1			
FMI	0.659	0.443–0.979	.039
LMI	0.485	0.307–0.767	.002
Model 2			
FMI	0.653	0.430–0.991	.045
LMI	0.480	0.297–0.774	.003
Model3			
FMI	0.745	0.444–1.251	.299
LMI	0.450	0.311–0.940	.029

Model1-unadjusted. Model 2-adjusted for diabetes duration, age, HbA1c, diabetic microvascular complications (DR, DN, and DPN), smoking and alcohol use. Model 3- adjusted for factors listed in Model 2 plus BMD at the lumbar spine, femoral neck and total hip.

CI = confidence interval, FMI = fat mass index, HbA1c = hemoglobin A1c, LMI = lean mass index.

## 4. Discussion

In this study, we found that patients with normal BMDs had higher BMI, FMI, and LMI than those diagnosed with osteoporosis and osteopenia. Both FMI and LMI were positively correlated with all BMDs measured at the 3 sites after adjusting for age. When we removed the mechanical load effect of body weight on BMD, the correlations between BMD values and FMI became negative or irrelevant, while those between the lumbar spine, total hip, and LMI remained positive. When further analyzed using multiple linear regression, the positive relationship between lean mass and BMDs still existed, while that between fat mass and BMD was largely lost in the hip and spine, indicating that individuals with higher lean mass will be expected to have higher BMDs at all regional sites even after controlling for other variables.

Patients with osteoporotic fractures had lower LMI and FMI values than their non-osteoporotic counterparts. LMI was positively associated with the presence of osteoporotic fractures independent of all risk factors. However, the protective effect of FMI on fractures was diminished when BMD values were considered.

Taken together, these results suggest that lean mass plays a leading role in the maintenance of BMD as a component of body weight. Moreover, it may protect against osteoporotic fractures in a BMD-independent manner in patients with T2DM over 50 years of age. Under the action of mechanical load, fat mass is positively associated with BMD, especially at the femoral neck, thus reducing the risk of osteoporotic fractures.

These positive effects of lean mass on BMD are consistent with the results of a number of previous population-based studies.^[17,21,22]^ It seems possible that these results are due to increased passive load, and muscle-induced strain may affect bone modeling, density, and geometry.^[23]^

The association between fat mass and BMD is unclear. Some cross-sectional studies have found a positive correlation between fat mass and BMD in elderly men.^[24–27]^ In a longitudinal analysis, Bleicher found that fat loss was strongly associated with hip BMD loss in men who lost weight after a 2.2- year follow up.^[28]^ However, some studies have suggested that fat mass negatively affects BMD.^[16,17,29]^ In a study on the relationship between metabolic syndrome components and osteoporosis, fat mass was found to be positively correlated with femoral neck bone mineral density in male diabetic patients.^[30]^ We found an inconsistency mainly due to whether the mechanical load effect of body weight on bone mass was adjusted; the variety of ethnic groups they chose as well as the different covariates they take account of may also contribute to the discrepancy. In our study, the positive correlation between FMI and BMD also became negative when body weight was adjusted, implying that fat mass may affect bone mineral density through weight-bearing mechanisms such as higher gravitational mechanical load on the bone. Regardless of the body weight, this effect may be weakened or even reversed. Fat mass is also considered to contribute to an increase in BMD by increasing the levels of hormones such as leptin,^[31]^ insulin,^[32]^ and preptin^[33]^ which are known to induce bone growth and directly stimulate osteoblasts; thus, more pathological mechanisms should be investigated in the future.

As mentioned earlier, T2DM patients tend to have higher BMD and fat mass, and lower lean mass than the healthy population. For both fat mass and lean mass, which could affect bone mass through the mechanism of mechanical load, it is unclear which one is the dominant factor in maintaining bone mass. In our study, FMI was found to be only positively associated with the femoral neck, and lean mass was more strongly associated with BMD than with FMI in multiple regression analysis, which is in line with previous population-based studies.^[21,34,35]^ In a meta-analysis involving 44 studies, Ho-Pham et al^[36]^ confirmed a stronger impact of lean mass on bone mineral density. For the diabetic population, a study recruited middle-aged individuals with uncomplicated noninsulin requiring T2DM and found that only lean mass significantly predicted BMD in the total body, hip, and femoral neck in male patients.^[37]^ According to the above, lean mass still plays a leading role in the regulation of bone mineral density in male patients aged over 50 years, similar to that in the general population.

Moreover, LMI, but not FMI, was found to be positively associated with the presence of clinical fractures even after adjustment for BMDs, implying that lean mass has a protective effect on fractures. A registry-based cohort study found that loss in total body lean mass, but not fat mass, was associated with increased fracture risk, independent of other risk factors.^[38]^ Another study with a noninsulin requiring T2DM population demonstrated that lean mass is significantly associated with hip strength,^[39]^ which may independently prevent falls and fractures. Whether resistance exercises or other therapies that build lean mass could be interventions for bone loss or fracture in T2DM requires further observation.

We also found that HbA1c, an indicator of glycemic control, was negatively correlated with LMI and FMI. The lean mass results are consistent with those of previous studies. In a cross-sectional study that enrolled 1474 diabetic patients aged ≥ 50 years, Tang et al^[40]^ found that a higher HbA1c level was associated with a lower LMI. Park et al^[19]^ found that poor glycemic control (HbA1c > 8.0%) is associated with poorer muscle quality. A longitudinal cohort study reported that hyperglycemia is associated with persistent lower muscle strength with aging, and HbA1c levels could predict a decline in muscle mass and strength. Several inflammatory signaling pathways, including ATP-dependent ubiquitin-proteasome pathway, calpains, autophagy, and cell apoptosis, which could be caused by advanced glycation end products and insulin resistance, may lead to an association between HbA1c and lean mass.^[41]^ Further studies are needed to confirm whether better glycemic control can preserve the lean mass in patients with diabetes.

Fat mass could be positively or not correlated with HbA1c in previous studies,^[26,42]^ while in our study, FMI was negatively correlated with HbA1c. Patients with diabetes may develop insulin resistance or deficiency. When insulin is deficient, glucose metabolism is restricted and lipolysis increases, resulting in a decrease in body fat. In the case of insulin resistance, there is usually excess fat and abnormal fat distribution. Therefore, with the deterioration in blood glucose control, the change in fat mass is uncertain.

It was recently reported that osteoporotic fractures may be closely related to diabetic microvascular complications,^[43]^ as reduced blood flow may contribute to bone loss and fragility. In our study, we also found that the incidence of DPN in the fracture population was relatively high. One unanticipated finding was that neither HbA1c nor diabetes duration was associated with BMD at each site. These results are in contrast to those of previous studies.^[19,44]^ The characteristics of poor glycemic control and a long diabetic course in these hospitalized patients may attenuate this impact.

The present study had some limitations. First, our study enrolled a relatively small sample, which has a limitation of temporal causality. Thus, prospective studies with larger samples that examine the association between body composition and the incidence of osteoporosis and fractures are required. Second, we included only subjects who visited our hospital for the evaluation and treatment of diabetes mellitus and osteoporosis. Therefore, the subjects enrolled in the present study might not be representative of Chinese diabetic male patients, especially young men. Third, we measured BMD and body composition using DXA, which is a noninvasive and effective method that allows separation of body mass into bone mass, fat mass, and lean mass; however, DXA-measured BMD values do not account for bone dimensional changes or allow for separation of the cortical and trabecular bone compartments. Finally, we did not investigate precise associations of BMD with regional fat or lean mass, which will be the focus of our future work.

## 5. Conclusion

Our study revealed that lean mass may predict bone mineral density at the lumbar spine, hip, and femoral neck and was positively associated with decreased osteoporotic fracture through a BMD-independent mechanism in male diabetic patients aged over 50 years. The effect of fat mass on bone may mainly rely on mechanical load to increase bone mineral density, thereby reducing the incidence of fractures. Whether increasing lean mass can protect patients from osteoporosis or osteoporotic fractures requires further investigation. Moreover, the role of fat mass with different distribution in bone metabolism requires further study.

## Author contributions

**Formal analysis:** Chuchen Meng.

**Investigation:** Chuchen Meng, Dan Zhao.

**Supervision:** Xin-Hua Ye.

**Writing – original draft:** Chuchen Meng.
